# Network Structure of Post-Traumatic Stress and Social/Emotional/Behavioral Difficulties in a Post-Earthquake Child and Adolescent Sample

**DOI:** 10.3390/ejihpe15110225

**Published:** 2025-10-31

**Authors:** Alberto Misitano, Febe Geddo, Annalisa Oppo, Alice Barbieri, Elena Righi, Ernesto Caffo, Barbara Forresi

**Affiliations:** 1 Trauma, Resilience, and Adjustment Investigation (TRAIL) Lab, Sigmund Freud University, 20143 Milan, Italy; geddo.ext@milano-sfu.it (F.G.); a.oppo@milano-sfu.it (A.O.); barbieri.ext@milano-sfu.it (A.B.); b.forresi@milano-sfu.it (B.F.); 2 Contextual Behavioral Science CBS-SFU Lab, Sigmund Freud University, 20143 Milan, Italy; 3 Istituto Europeo per lo Studio del Comportamento Umano (IESCUM), 43100 Parma, Italy; 4 Department of Biomedical, Metabolic and Neural Sciences, Università di Modena e Reggio Emilia, 41125 Modena, Italy; elena.righi@unimore.it (E.R.); ernesto.caffo@unimore.it (E.C.)

**Keywords:** children, adolescents, PTSD, trauma, earthquake, natural disasters, network analysis

## Abstract

Following natural disasters, children and adolescents are particularly vulnerable to the onset and persistence of post-traumatic stress symptoms, which can significantly affect developmental trajectories and mental health. Although PTSD networks have been extensively studied in adults, less is known about youth, and no previous studies have examined how PTSD clusters relate to social, emotional, and behavioral difficulties (SEBD). This study applied network analysis to examine how PTSD clusters relate to psychosocial problems in a large sample of trauma-exposed youth. A total of 635 Italian children and adolescents (M_age_ = 11.19 years, SD = 1.43; 51.5% male), exposed to the 2012 Emilia-Romagna earthquake, completed the UCLA PTSD-RI and the Strengths and Difficulties Questionnaire. Network estimation and centrality indices were computed for the overall sample. Network Comparison Tests assessed differences by gender, age group, and proximity to the epicenter. Emotional problems and Increased Arousal emerged as the most central nodes, whereas Peer Problems were consistently peripheral. Gender differences were significant, whereas no differences were detected by age; moreover, youth living closer to the epicenter exhibited a more densely connected network. Despite its limitations, the study identifies co-occurrence patterns between PTSD clusters and specific SEBD, outlining clinical implications that warrant further investigation.

## 1. Introduction

Globally, about 175 million young people each year experience natural disasters such as floods, earthquakes, hurricanes, and droughts (Lai et al., 2020). Although natural disasters expose individuals of all ages to life-threatening events and widespread disruption, elevating the risk of PTSD (Benjet et al., 2016; Keya et al., 2023; Newnham et al., 2022; Nolting et al., 2025), children and adolescents are especially vulnerable to both its onset and persistence (Alisic et al., 2014; Lai et al., 2020; Meiser-Stedman et al., 2019; C.-W. Wang et al., 2013; Woolgar et al., 2022). Reported prevalence of post-traumatic stress symptoms (PTSS) in child and adolescent survivors ranges from 1% to 95% following disasters (C.-W. Wang et al., 2013). According to a recent meta-analysis, the pooled prevalence of PTSD among child and adolescent survivors of earthquakes and floods was 19.2% (95% CI: 18.6–19.7), 30.0% (95% CI: 29.5–30.6), 24.4% (95% CI: 23.4–25.4), and 20.4% (95% CI: 19.1–21.7) across the first, second, third, and fourth six-month intervals post-disaster, respectively, with a significantly higher prevalence in girls (Rezayat et al., 2020).

According to a developmental psychopathology framework, individual vulnerabilities, life experiences, and contextual factors interact to shape the onset, presentation, and functional impact of PTSD (R. S. Pynoos et al., 1999). Children and adolescents may display PTSD symptoms differently from adults, depending upon the developmental stages, the level of cognitive and emotional maturity, previous psychological vulnerabilities and social support (Bastien et al., 2020; Gkintoni et al., 2024; Helpman et al., 2015). At the same time, PTSD can simultaneously affect multiple domains of a young person’s life, academic performance, interpersonal relationships, and overall well-being (R. S. Pynoos et al., 1999). Beyond PTSD, children exposed to traumatic events may also show poor emotion regulation, attention problems, irritability, anger, and conduct problems (Briggs-Gowan et al., 2016; McLaughlin & Lambert, 2017). Trauma-exposed youth often display emotion-regulation difficulties, such as disruptions in the processes of fear acquisition and extinction, difficulties in discriminating between safe and unsafe environments, heightened reactivity to potential threats, and inability to disengage from negative emotional content (McLaughlin & Lambert, 2017). While these behavioral changes may serve to protect them in dangerous environments (McLaughlin & Lambert, 2017), they can also have profound developmental consequences on academic performance, peer integration, and long-term mental health (A. Goodman et al., 2010; Cicchetti & Toth, 1995; Kim & Cicchetti, 2010). Previous studies show that in youths exposed to potentially traumatic events, PTSD symptoms often co-occur with emotional, behavioral, and social difficulties measured, for example, by the Strengths and Difficulties Questionnaire (Crum et al., 2018; El-Khodary & Samara, 2020; Jonkman et al., 2013; Juen et al., 2024; Mann et al., 2015; McDermott et al., 2005; Villalta et al., 2020). However, the complex connections among PTSD clusters (Russell et al., 2017) and social/emotional behavioral difficulties (SEBD) remain poorly understood, particularly whether these difficulties relate uniformly to all PTSD clusters or show cluster-specific associations.

In this regard, the network approach is being increasingly used to examine the interrelationships among psychological variables across various clinical conditions (Borsboom et al., 2021), including PTSD (Birkeland et al., 2020; Isvoranu et al., 2021). In this approach, the connections (i.e., bridges) between variables (i.e., nodes) constitutes the psychopathological phenomenon itself, without positing any underlying cause: clinically relevant information is gathered inspecting the overall network structure, the connections between variables, and the relative position (i.e., centrality) of each variable in the network (Borsboom et al., 2021; McNally et al., 2015). Therefore, the network approach is well-suited for exploring the roles of multiple variables within a complex single system (Borsboom et al., 2021; Misitano et al., 2024) such as psychopathology (Borsboom et al., 2021; Spiller et al., 2024) and, more specifically, post-traumatic responses (Galatzer-Levy & Bryant, 2013; Spiller et al., 2024).

Regarding PTSD, two recent systematic reviews (Birkeland et al., 2020; Isvoranu et al., 2021) have highlighted the complex network structure of PTSD symptoms. Nevertheless, these studies were predominantly on adult samples. Russell et al. (2017) pioneered the field by examining the network structure of DSM-IV PTSD symptoms in youth affected by Hurricanes Katrina and Gustav. The study revealed both cross-age associations, in which avoidance and physiological reactivity were consistently linked across age groups, and age specific associations, with numbness and amnesia appeared specifically in children (Russell et al., 2017). Subsequent studies on post-earthquake adolescent samples highlighted the central role of cross- age symptoms, with avoidance (Rossi et al., 2022), intrusive symptoms (Xu et al., 2023), intrusive thoughts and flashbacks (Chen et al., 2025), emerging as the most central variables in PTSD networks. Taken together, these findings indicate that PTSD symptom networks in youth include both core cross-age associations and age-specific features that may be more salient in childhood.

Recent literature adopted network analysis to investigate the relationships between PTSD and co-occurring problems in youth. For instance, (Vázquez et al., 2024) found that sleep disturbance and physiological arousal were key bridge symptoms between exposure to a natural disaster and substance use in children and adolescents. Other studies (Qi et al., 2023; Xu et al., 2023) showed that PTSD dysphoric symptoms were a strong bridge to depression in adolescents exposed to earthquakes. Collectively, these studies highlighted the usefulness of the network approach to gain fine-grained, clinically relevant information regarding the complex relationships between PTSD clusters and other trauma-related symptoms. However, none of previous studies explored the network structure of PTSD clusters in conjunction with SEBD in youth, even though these dimensions are key determinants to capture the complexity of adaptation of youth to potentially traumatic events such as natural disasters (McLaughlin et al., 2012; Weems & Overstreet, 2008). By identifying which PTSD clusters are most strongly associated with emotional, conduct, and peer-related problems, researchers may help clarify the pathways through which post-traumatic stress is maintained or contributes to broader developmental difficulties.

Building on prior research in the same sample (Caffo et al., 2005; Forresi et al., 2020, 2021), the present study adopts the network approach focusing on the interaction between PTSD-related clusters and SEBD in children and adolescents exposed to the 2012 Emilia-Romagna earthquake. Inspired by Kurt Lewin’s Gestalt principle that “the introduction of an element modifies the entire system,” it examines how incorporating nodes for emotional, behavioral, and social problems into a PTSD network alters its overall structure and connectivity.

Specifically, drawing on the theoretical background and the empirical evidence summarized above, in the following sections we first describe the study design and the analytic strategy adopted to examine, using the network approach, the relationship between PTSD clusters and SEBD in a sample of Italian children and adolescents two years after being exposed to the 2012 Emilia Romagna earthquake. Secondary objectives included comparing network structures by sex, age group, and proximity to the disaster’s epicenter. We then present the key findings, followed by a discussion that integrates the results with prior literature, highlights theoretical and clinical implications, and notes limitations and directions for future research.

Given the absence of prior network analyses integrating PTSD clusters and SEBD, no a priori hypotheses were formulated regarding age- or gender-specific differences in network structure. However, it was expected that certain clusters would emerge as more influential within the overall network.

## 2. Materials and Method

### 2.1. Participants and Procedure

In this study, previously published data are re-examined through a novel network approach (Forresi et al., 2020). The original sample was composed of 682 children and adolescents, exposed to the 2012 Emilia-Romagna earthquake and assessed 18 months after the event. To select the sample, the Province of Modena (Northern Italy) was divided into two different areas: an ‘Earthquake Area’ (EA), near the epicenter, including the plain zones of the Province most affected by the earthquake; and a ‘Control Area’ (CA), including plain zones and hills less directly affected by the earthquake, where no damages to buildings or individuals occurred (Forresi et al., 2020).

As participants with missing data were excluded, the final study sample included 635 children and adolescents recruited from ten different schools randomly selected. This research was approved by the Ethics Committee of Modena (Protocol No. 268/12). Prior to conducting the assessment, the School Principal and School Board gave their approval. In addition, parents were provided with a comprehensive explanation of the study and gave written informed consent. The study’s procedures were clarified to the students, and only those who provided their personal consent were included. For further information on the recruitment procedure see Forresi et al. (2020).

### 2.2. Measures

Children and adolescents received a comprehensive assessment protocol, including the following components:

- Exposure: an ad hoc questionnaire was developed to gather demographic information and data on the extent of exposure to the earthquake. It included questions about personal, family, or friends’ injuries or deaths, property damage, displacement. The questionnaire used a binary scale (“*yes*” = 1, “*no*” = 0) to assess each item (Forresi et al., 2020). For primary school children, the interviewer administered the questionnaires, while secondary school children and adolescents completed the questionnaires on their own.
- UCLA Post-Traumatic Stress Disorder Reaction Index (PTSD-RI; R. Pynoos et al., 1998): This instrument, designed for children and adolescents aged 7–17 who have experienced a potentially traumatic event, screening for trauma exposure and PTSD symptoms as defined by DSM-IV. It is widely used and well-validated (Steinberg et al., 2004). As described in the scoring sheet provided by the developers of the PTSD-RI, the questionnaire includes 20 items rated on a five-point Likert-type scale, allowing calculation of severity scores of three symptoms clusters (Reexperiencing, Avoidance, and Increased Arousal) as well as an overall total severity score (R. Pynoos et al., 1998). High internal consistency has been reported with a Cronbach’s alpha of 0.90, and test–retest reliability of 0.84 (Steinberg et al., 2004). Although no formal Italian validation exists, the questionnaire was translated following rigorous standards, including independent back-translation (Goenjian et al., 1995). Within the present sample, the analysis revealed a Cronbach’s alpha of 0.84 reflecting a strong internal consistency, comparable to the data from the original instrument.
- Strengths and Difficulties Questionnaire (SDQ; R. Goodman, 1997; Italian version: Tobia et al., 2013): The SDQ is a 25-item screening instrument for emotional and behavioral problems in children and adolescents, organized into five subscales of five items each: Emotional Problems, Conduct Problems, Hyperactivity–Inattention, Peer Relationship Problems, and Prosocial Behavior. Items are rated on a three-point Likert scale (“not true,” “somewhat true,” “certainly true”). An overall difficulty score (0–40) is obtained by summing the first four subscales (excluding Prosocial Behavior). Italian normative data are available (Tobia & Marzocchi, 2018), and the Italian SDQ has demonstrated solid psychometric properties, with subscale Cronbach’s alphas ranging from 0.73 to 0.89 (Tobia & Marzocchi, 2018). In the present sample, internal consistency was acceptable (Cronbach’s α = 0.71).

### 2.3. Statistical Analysis

IBM SPSS Statistics (v. 29 for macOS; IBM Corp., 2023) was used for all descriptive statistical analyses, while R (v. 4.4.1 for Windows; R Core Team, 2024) was employed for network analyses, adhering to the standard guidelines outlined by Epskamp and Fried (2018). To examine the relationship between PTSD clusters and SEBD, the networks were composed of eight nodes: the three PTSD symptoms clusters of the UCLA PTSD-RI (re-experiencing, avoidance/numbing, and hyperarousal, according to DSM-IV) and the five subscales of the SDQ (Emotional Problems, Conduct Problems, Hyperactivity, Peer Problems, Prosocial Behavior). Although analyzing each individual symptom would provide more detailed information, we grouped symptoms into clusters to ensure stable, reliable, and interpretable network estimates given the modest sample size (an approach used in prior studies; e.g., Armour et al., 2020; Greene et al., 2020; Liebman et al., 2021; Rønning et al., 2025; Weiss et al., 2020) and recommended for accurate estimation with limited data (Epskamp et al., 2018).

Network analyses were estimated using the Extended Bayesian Information Criterion Graphical Least Absolute Shrinkage and Selection Operator (EBICglasso), which is a regularization method that estimates partial correlations among all variables in the network (Friedman et al., 2014). Centrality indices were estimated to evaluate the relative importance of each node within the network. In this study, the following indices were considered: strength (i.e., how well a node is directly connected to other nodes), closeness (i.e., how close a node is to all other nodes in the network), and betweenness (i.e., the frequency with which the node is found in the shortest path between two other nodes), and expected influence, which takes the sign of edge weights into account (Borsboom et al., 2021). The higher the value, the higher the centrality of each node. Then, stability coefficients of centrality indices were explored, with values > 0.5 suggesting ideal stability (Epskamp et al., 2018). 1000 non-parametric bootstrapped samples were used to explore the accuracy of edge weights in the network with 95% confidence intervals and to test for significant differences between edges. The following packages were used to estimate the networks: *qgraph* (Epskamp et al., 2012), *bootnet* (Epskamp et al., 2018), *mgm* (Haslbeck & Waldorp, 2020), *corpcor* (Schäfer & Strimmer, 2005), *networktools* (Jones, 2017), and *igraph* (Csárdi et al., 2025). Additionally, the *NetworkComparisonTest* (NCT) package (van Borkulo et al., 2023) was used to explore potential network differences according to binary gender, primary and secondary school attendance and proximity to seismic crater, considering the following tests: Network Invariance Test (to identify significant differences in the maximum edge strength in the networks), Global Strength Invariance Test (to pinpoint significant differences in the overall connectivity), Centrality Invariance Test (to determine whether a node identified as “central” in the network remains central in subgroup networks), and the Edge Invariance Test (to explore edge-specific differences in networks with significant network invariance) (van Borkulo et al., 2023).

## 3. Results

### 3.1. Descriptives

Overall, the sample included 635 participants with a mean age of 11.19 ± 1.43 years (range: 8–15); of these, 327 (51.5%) were male and 308 (48.5%) were female. The sample included 222 (35%) primary school students and 413 (65%) secondary school students, with 405 (63.8%) and 230 (36.2%) in the earthquake and in the control area, respectively. None of the children or adolescents engaged with mental health services before the assessment. Descriptive statistics for clinical variables, including PTSD symptom clusters and SEBD, are provided in Table 1.

### 3.2. Network Analysis

#### 3.2.1. Overall Sample

**Figure 1:**
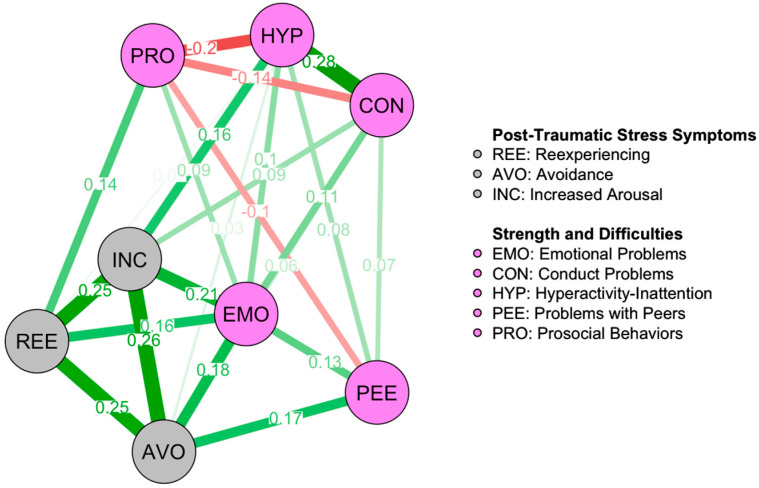
Overall Network Graph. ***Note.*** Network model of PTSD clusters and SEBD in youth exposed to the 2012 Emilia-Romagna earthquake. The model was regularized with the Extended Bayesian Information Criterion (EBIC) using Graphical Least Absolute Shrinkage and Selection Operator (EBICglasso). Green lines represent positive partial correlations between nodes (i.e., variables), while red lines represent negative partial correlations. Values reported on each line represent the partial correlation coefficient: higher values correspond to thicker lines. The sparsity of the network, indicating the proportion of zero-valued connections, is 0.143.

The network analysis conducted on the overall sample (Figure 1) revealed 24 non-zero edges out of possible 28, indicating a sparsity of 0.143. Additionally, the confidence interval of the accuracy of edge weights (Figure S1) is relatively narrow, suggesting interpretability of the network. Differences between each edge in the network are reported in Figure S2. Stability analyses of centrality metrics (Figure S3) indicate that strength, expected influence, and closeness are interpretable, as all exhibit values greater than 0.5. Conversely, betweenness has proven to be significantly more unstable, as frequently reported in the literature (Borsboom et al., 2021). Therefore, the evaluation of centrality will primarily focus on the other three indices.

As reported in Figure 1, nodes representing PTSD clusters were highly interconnected, as well as nodes representing the subscales of the SDQ: only Emotional Problems showed direct connections with each of the PTSD clusters, with differences among them not statistically significant. Additionally (Table 2), the three most central nodes were Emotional Problems, Increased Arousal, and Avoidance, while Peer Relationship Problems and Prosocial Behaviors were the least central nodes.

#### 3.2.2. Network Comparison According to Gender

**Figure 2:**
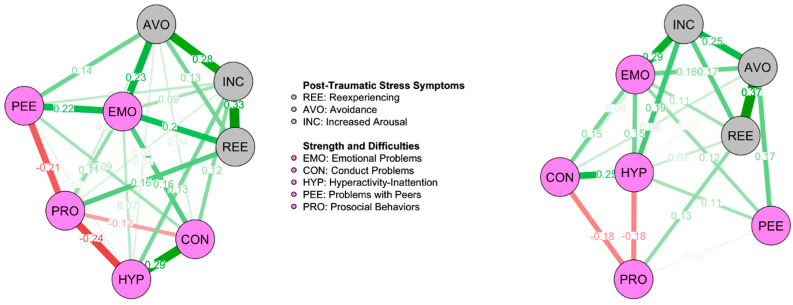
Networks for Males (**left**) and Females (**right**). ***Note.*** Network model of PTSD symptoms and general psychopathology in male (**left**) and female (**right**) youth exposed to the 2012 Emilia-Romagna earthquake. The model was regularized with the Extended Bayesian Information Criterion (EBIC) using Graphical Least Absolute Shrinkage and Selection Operator (EBICglasso). Green lines represent positive partial correlations between nodes (i.e., variables), while red lines represent negative partial correlations. Values reported on each line represent the partial correlation coefficient: higher values correspond to thicker lines. The sparsity of the network, indicating the proportion of zero-valued connections is 0.107 and 0.214, respectively.

The male network (Figure 2) was denser (25/28 possible non-zero edges; sparsity = 0.107) than the female network (Figure 2; 22/28 possible non-zero edges; sparsity = 0.214), suggesting a higher number of connections between nodes. However, the Global Strength Invariance Test was not significant (S = 0.331, *p* > 0.05), suggesting that the overall connectivity is comparable between the two networks.

Additionally, the Network Invariance Test showed statistical significance (M = 0.237, *p* = 0.045), suggesting the following edge-specific differences: Reexperiencing-Avoidance (stronger in females; E = 0.237, *p* < 0.001), Increased Arousal-Emotional Problems (stronger in females; E = 0.192, *p* < 0.05), and Peer Problems-Prosocial Behaviors (stronger in males; E = −0.188, *p* < 0.05).

Regarding centrality (Tables S1 and S2), Emotional Problems and Increased Arousal (followed to a lesser extent by Avoidance) were the most central nodes in the male network, while Emotional Problems and Avoidance (followed to a lesser extent by Increased Arousal) were the most central in the female network. Peer Problems was the least central node in both networks. Nevertheless, the Centrality Invariance Test revealed only significant differences in centrality for Peer Problems (strength higher in males than in females; C = 0.341, *p* < 0.05) and Hyperactivity (expected influence higher in females; C = 0.289, *p* < 0.05).

#### 3.2.3. Network Comparison According to Age Group

The network of primary school students (Figure S4) was slightly denser (25/28 non-zero edges; sparsity = 0.107) than the secondary school students’ network (Figure S5; 24/28 non-zero edges; sparsity = 0.143). Comparisons between the networks were not significant, suggesting no meaningful differences in network maximum edge strength (M = 0.222), in the overall connectivity (S = 0.059), or specific edges (all *p*s > 0.05). In the primary school students’ network, Emotional Problems and Increased Arousal (followed by Reexperiencing) showed the highest centrality metrics, while Avoidance and Increased Arousal (followed by Emotional Problems) showed the highest centrality metrics in the secondary school student network (Tables S3 and S4); nevertheless, no statistically significant differences between centrality indices were found among the two networks (all *p*s > 0.05).

#### 3.2.4. Network Comparison According to Proximity to the Epicenter

**Figure 3:**
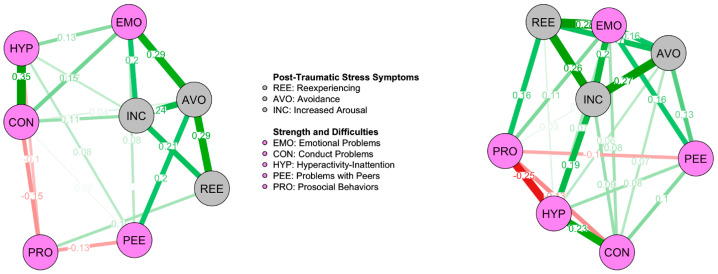
Networks for Control Area (**left**) and Earthquake Area (**right**). ***Note.*** Network model of PTSD clusters and SEBD in youth from the Control (**left**) and the Earthquake (**right**) Area. The model was regularized with the Extended Bayesian Information Criterion (EBIC) using Graphical Least Absolute Shrinkage and Selection Operator (EBICglasso). Green lines represent positive partial correlations between nodes (i.e., variables), while red lines represent negative partial correlations. Values reported on each line represent the partial correlation coefficient: higher values correspond to thicker lines. The sparsity of the networks, indicating the proportion of zero-valued connections, is 0.286 and 0.143, respectively.

Participants closer to the epicenter showed a denser network (Figure 3; 24/28 non-zero edges; sparsity = 0.143) than participants living more distant from it (Figure 3; 20/28 non-zero edges; sparsity = 0.286). The network of participants closer to the epicenter showed higher maximum edge strength (M = 0.277, *p* < 0.05), while the sum of edge strengths was not statistically different between the networks (S = 0.467, *p* > 0.05). Regarding the edges, the Reexperiencing-Emotional Problems was significantly stronger for the network of participants near the seismic crater (E = 0.277, *p* < 0.01).

Regarding centrality (Tables S5 and S6), while Emotional Problems, Increased Arousal and Reexperiencing showed highest centrality in the network of participants closer to the crater; in the network of participants farther to the epicenter, Emotional Problems, Avoidance and Increased Arousal showed the highest centrality metrics. Nevertheless, there were no statistically significant differences in centrality according to the Centrality Invariance Test (all *p*s > 0.05).

## 4. Discussion

This study aimed to investigate the network structure of **PTSD** clusters and associated emotional, behavioral and social difficulties (SEBD) in Italian children and adolescents 18 months after exposure to the 2012 Emilia Romagna earthquake, using a network analysis. Differences based on binary gender, age, and proximity to the epicenter were also explored. To our knowledge, this is the first study to integrate PTSD symptom clusters with a broader range of SEBD (measured by the SDQ) into a single network model in youth exposed to an earthquake.

In the full sample, although centrality metrics varied slightly across groups, Emotional Problems and Increased Arousal consistently emerged as the most central nodes, displaying the highest levels of expected influence centrality. Unlike earlier studies on PTSD networks in children and adolescents—that did not identify hyperarousal as central (Russell et al., 2017), despite its prominence in adult samples (Bryant et al., 2017; McNally et al., 2015)—the present findings indicate that Increased Arousal held a consistently central position across all networks, with strong direct connections with both Reexperiencing and Avoidance clusters. This pattern supports the tightly interconnected nature of core PTSD features, as also highlighted in more recent work (Scharpf et al., 2023). Furthermore, the centrality of Hyperarousal in a sample assessed 18 months after the earthquake may also reflect its persistence over time, in accordance with prior evidence that this cluster tends to be the most resistant to change, even following treatment (Larsen et al., 2019).

The present study also contributes to the literature by demonstrating the centrality and strong association between Increased Arousal and Emotional Problems in maintaining symptom networks among youth. Although the cross-sectional design does not allow for causal inferences, this finding is particularly noteworthy, as it is consistent with the hypothesis that emotional dysregulation may serve as a key mechanism linking arousal-related stress responses to broader psychosocial difficulties in trauma-exposed children. This result is consistent with previous work in adult samples, including a temporal network analysis showing that alterations in the arousal cluster had the strongest bridging links with negative emotions (Greene et al., 2020), and a cross-sectional network analysis linking arousal to positive emotion dysregulation (Weiss et al., 2020).

Although the cross-sectional design and the exploratory nature of the analyses which preclude any inference about directionality, the strong link between Hyperarousal and Emotional Problems may reflect a mutually reinforcing dynamic, whereby physiological hyperactivation increases vulnerability to emotional difficulties, which in turn may maintain or intensify arousal-related responses. This bidirectional interaction has been proposed in both developmental trauma frameworks (R. S. Pynoos et al., 1999) and, consistent with this interpretation, previous research has shown that traumatic experiences can alter threat-processing mechanisms (McLaughlin & Lambert, 2017), and that children and adolescents with trauma histories frequently exhibit atypical physiological responses to perceived threat (McLaughlin et al., 2014). These findings are also in line with inhibitory learning models of trauma-related psychopathology, which suggest that disruptions in the extinction of fear responses may sustain pathological arousal over time (McLaughlin & Lambert, 2017; Lissek & van Meurs, 2015). Although these mechanisms remain understudied in pediatric populations, emerging evidence indicates that youth with PTSD often show intact fear acquisition but impaired extinction of conditioned fear responses, potentially contributing to chronic hyperarousal and prolonged distress in trauma-related contexts (Grasser et al., 2023; McGuire et al., 2016). Future prospective studies are nevertheless needed to better understand these dynamics.

Across the full sample, Peer Problems and Prosocial behaviors consistently ranked as the least central node. The peripheral placement of these social functioning indicators may reflect multiple, non-mutually exclusive explanations. On the one hand, in the months following a natural disaster, difficulties and strengths in peer relationships may not be directly embedded in the core post-traumatic symptomatology, but instead maintained via alternative pathways, a hypothesis warranting further longitudinal examination (Weems & Overstreet, 2008). Supporting this, a more detailed examination of the network structure in the present study highlights the potential bridging role of Emotional Problems (along with Reexperiencing) in linking Hyperarousal to Peer Problems, suggesting that dysregulated emotions may serve as pathways through which physiological distress manifests in the social domain.

However, although traditionally centrality measures have been suggested to reflect the relative importance of each node in the network, with more central nodes as potential treatment targets (Borsboom et al., 2021), recent works highlighted that centrality is not always a good proxy for causal inference: for certain networks, in fact, peripheral nodes (i.e., the nodes with the lowest centrality indices) may be more important in determining system behavior (Borsboom et al., 2021; Dablander & Hinne, 2019; Quax et al., 2013; Spiller et al., 2020). Such perspective would be in line with findings showing that peer-relationship difficulties can exacerbate Avoidance and Hyperarousal, thus contributing to the maintenance of PTSD in youth (La Greca et al., 2001). Given the cross-sectional nature of the present study, causal inferences cannot be drawn, and no definitive conclusions can be made regarding the relative importance of central versus peripheral nodes. Future longitudinal and experimental research should address the clinical utility of monitoring and targeting Emotional Problems, being related to both Increased Arousal, and Peer Problems in the treatment of PTSD in children and adolescents.

Additionally, a positive association was observed between Emotional Problems and Prosocial behavior in participants in the Earthquake Area, suggesting that emotional dysregulation may predispose children to engage in prosocial actions in high-exposure contexts. These findings align with three complementary frameworks within the trauma and prosociality literature. First, the “Altruism Born of Suffering” model posits that adverse experiences can enhance empathic concern and motivate individuals, including children, to help others (Staub & Vollhardt, 2008). Second, the Post-traumatic Growth perspective suggests that trauma may foster deeper interpersonal connections and altruistic behavior as part of adaptive psychological growth (Tedeschi & Calhoun, 1996). Finally, the Negative State Relief model proposes that children experiencing heightened emotional distress may engage in prosocial acts as a way to alleviate their own discomfort (Cialdini et al., 1973). In the specific context of Emilia-Romagna, these dynamics may also be reinforced by the region’s long-standing cultural emphasis on social solidarity and community support, which historically fosters collective responses to adversity.

Despite comparable overall network connectivity across genders, and no statistical differences in node centrality, edge-wise analyses showed significant differences, suggesting a stronger association in males between Hyperarousal and Emotional Problems, as well as between Peer Problems and Prosocial Behavior (a negative link). Conversely, Reexperiencing and Avoidance appeared to be more closely connected in females. While we can only speculate on the nature of this difference, it might be that intrusive thoughts and avoidance are more central in the PTSD response of young females. These differences echo prior reports of gender-differences (Cao et al., 2019; Greene et al., 2020) and gender-divergent coping styles, with boys more likely to externalize distress through social conflict, and girls to internalize it via avoidance (Gauthier-Duchesne et al., 2017; Olff, 2017). Tentatively, the pattern observed in men may also reflect the influence of societal gender norms and roles on how distress is perceived and expressed. Further studies are needed to clarify the role of gender in post-disaster responses.

Primary and secondary school networks shared remarkably similar structures and overall connectivity, suggesting a developmental consistency across age groups. Additionally, non-significant differences emerged in symptom cluster edges. This finding differs from previous observations (Russell et al., 2017), showing meaningful variability across age in the symptom network, but is in line with the meta-analysis by Furr et al. (2010), finding no evidence of a significant age effect in pediatric PTSD after disasters. A first possible explanation is that when traumatic exposure is shared and homogeneous (as in the case of large-scale natural disasters) the variability introduced by age-related developmental processes may be attenuated. In such contexts, the magnitude of the trauma may override subtle maturational differences in emotional and cognitive regulation. In the present study, however, in the present study, a plausible explanation is that the assessment occurred 18 months after the earthquake, a period during which initial age-related differences may have faded.

The comparison of networks based on proximity to the seismic epicenter revealed the most pronounced significant difference in network structure, suggesting that direct exposure to the disaster may shape the interplay among post-traumatic symptoms in youth residing nearer to the seismic crater exhibited a denser network. These findings suggest that, even 18 months after the disaster, youth living nearer the epicenter exhibit more densely interconnected PTSD and SEBD cluster networks than those in more distant areas. Such tightly knit networks are especially vulnerable to hysteresis, a phenomenon described as self-reinforcing loops of symptom activation that persist even after external triggers have disappeared (Borsboom, 2017; McNally et al., 2017), which may contribute to the persistence of PTSD, in line with dose–response models of trauma exposure in youths (Furr et al., 2010; La Greca et al., 2002; Norris et al., 2002a, 2002b; Pine et al., 2005). This finding is consistent with previously published data on the same sample by the research group (Forresi et al., 2020), in which regression analyses identified severity of exposure (i.e., personal injury and bereavement) as one of the strongest predictors of children and adolescent post-traumatic symptoms and psychosocial difficulties.

Although the stability in centrality rankings suggests that Increased Arousal and Avoidance consistently represent core PTSD processes across exposure levels, the stronger association between Reexperiencing and Emotional distress observed in the Earthquake Area points to qualitative differences in how clusters interact under higher levels of traumatic exposure. This finding may indicate that, for children and adolescents located close to the epicenter, intrusive memories are more tightly coupled with affective dysregulation, possibly due to more vivid, emotionally intense, and contextually embedded trauma representations (Brewin et al., 2010; Engelhard et al., 2009). Such strengthening of symptom-to-symptom connections, without changes in overall node centrality, underscores the importance of examining not only which clusters are central, but also how they relate to one another within different exposure contexts. In line with network theory, this pattern suggests that symptom interactions, rather than symptom prevalence or intensity alone, may vary meaningfully by trauma severity, shaping the phenomenological experience of PTSD and informing context-sensitive interventions.

### Strengths and Limitations

This study presents several strengths that contribute to the growing body of research on PTSD symptom networks in children and adolescents. First, the use of network analysis provides a granular understanding of how PTSD clusters interact, moving beyond traditional categorical approaches and offering insights into key clusters connectivity patterns. Second, the study includes a well-characterized sample of children and adolescents exposed to a real-life natural disaster, which enhances the ecological validity of our findings. Third, the application of Network Comparison Tests (NCTs) allows for the examination of age, gender, and exposure-related differences, which are crucial for identifying population-specific symptom structures.

However, some limitations must be acknowledged. First, the cross-sectional nature of the study precludes conclusions about the directionality of symptom clusters interactions within the network. As such, the present network model provides a valuable snapshot of cluster interconnections at a specific point in time but cannot determine whether certain clusters function as drivers or consequences within the network. Future studies incorporating repeated assessments or temporally structured data would be better suited to explore how these interactions unfold and potentially shift during the course of post-traumatic adaptation. This is particularly relevant to another limitation of the study, namely the timing of data collection. Because participants were assessed 18 months after the earthquake, future prospective investigations that capture responses before the disaster, in the aftermath, and across both the short and long term after the trauma are warranted to deepen our understanding of trauma-related trajectories and the influence of additional life events such as new stressors, social changes, or ongoing recovery challenges.

Second, the present study relies exclusively on self-report measures, i.e., the *Strength and Difficulties Questionnaire* (SDQ) and the *UCLA PTSD Reaction Index* (UCLA PTSD-RI). While such instruments have demonstrated solid diagnostic utility and feasibility in post-disaster contexts (Steinberg et al., 2004), this approach would benefit from integration with additional assessment methods, such as clinical interviews and qualitative approaches. These complementary strategies would offer a more nuanced understanding of symptom meaning, expression, and interpersonal dynamics, particularly in diverse or trauma-exposed populations.

Additionally, the UCLA PTSD-RI has not been formally validated in the Italian context and remains anchored to the DSM-IV. To build on the exploratory nature of the present study, future research should employ validated instruments to assess PTSD clusters in children and adolescents according to the DSM-5 (Friedman, 2013; Kilpatrick et al., 2013) and the DSM-5-TR diagnostic criteria (American Psychiatric Association, 2022). The revised criteria introduce an additional diagnostic domain that includes persistent negative thoughts, pervasive negative emotions, and reckless or destructive behavior, which were not captured in the present study. Future network analyses including the revised PTSD clusters may reveal different patterns of connectivity and centrality.

Furthermore, the sample is restricted to an Italian population, potentially limiting the generalizability of the findings to other cultural contexts. Cross-cultural replications across diverse natural-disaster contexts and sociocultural environments will shed light on the generalizability of network configurations and reveal how cultural norms might modulate symptom connectivity in youth (Betancourt et al., 2013).

Notwithstanding the limitations described above, mapping the connections between specific PTSD symptom clusters and the five SDQ dimensions remains a valuable direction for future research. Psychosocial difficulties captured by the SDQ are well-established predictors of academic performance, peer integration, and long-term mental health outcomes (A. Goodman et al., 2010). Clarifying how distinct PTSD symptom clusters relate to these broader, developmentally salient impairments could enhance prognostic accuracy and inform early, targeted interventions aimed at supporting psychosocial functioning in youth, across domains such as peer relationships. To further refine this approach, finer-grained developmental analyses are needed to distinguish symptom constellations specific to early childhood, middle childhood, and adolescence. Equally important is the integration of contextual analyses that consider sociocultural, educational, and environmental factors, which shape how symptoms are experienced, expressed, and interpreted across different settings.

With regard to clinical applications, the robust connections between Increased Arousal and Emotional Problems suggest that hyperarousal is closely linked to emotional dysregulation and may reflect the activation of evolutionarily older fear circuitry, which operates automatically and with limited involvement of higher-order cognitive control. Although interventions that directly target hyperarousal may lead to downstream improvements in Emotional Problems, current evidence suggests that this PTSD symptom cluster often shows the weakest response to standard treatments. For instance, first-line cognitive behavioral therapies (such as CBT, CPT, and exposure-based approaches) have demonstrated larger effects in reducing avoidance and intrusive symptoms than in attenuating hyperarousal (Larsen et al., 2019; Schnurr & Lunney, 2015).

These findings point to the need for dedicated components that specifically address deficits in emotion regulation in youth, which could enhance the effectiveness of core PTSD treatments for youth. By directly targeting hyperarousal and related difficulties in extinction learning (McGuire et al., 2016), such interventions may help children and adolescents break maladaptive emotional cycles and support longer-term resilience. In line with findings from adult samples, improvements in hyperarousal and emotional functioning may also facilitate broader changes in psychosocial domains, including peer relationships, academic performance, and behavioral adjustment (Shnaider et al., 2014).

However, it is crucial to exercise caution when translating centrality findings into treatment recommendations. The interpretation of centrality indices in psychological networks, in fact, remains a topic of debate, as these measures may not always align with how clusters interact in real-world clinical settings (Bringmann et al., 2019). Focusing exclusively on high-centrality clusters may overlook other clinically significant aspects; therefore, interventions should be guided by both empirical evidence and clinical expertise.

## 5. Conclusions

This study contributes to the growing body of literature on PTSD and post-traumatic consequences in youth by highlighting the complex interplay among PTSD clusters and social, emotional, and behavioral difficulties in youth and by demonstrating how gender and contextual factors, such as proximity to the disaster epicenter, may shape these networks. By capturing these context-sensitive, symptom-level dynamics, network analysis offers a nuanced framework for refining developmental models of trauma response and informing targeted interventions. Rather than relying exclusively on diagnostic categories, this approach emphasizes specific constellations of clusters that may represent more precise and effective targets for clinical intervention in disaster-exposed children and adolescents.

## Figures and Tables

**Table 1:** Descriptives of the clinical variables.

Variable	Mean	SD	Min.	Max.	Skewness	Kurtosis
**UCLA PTSD-RI**						
**Reexperiencing**	3.30	3.544	0	19	1.313	1.328
**Avoidance**	5.10	4.119	0	23	0.986	0.505
**Increased Arousal**	5.60	3.281	0	18	0.820	0.647
**SDQ**						
**Emotional Problems**	2.42	2.205	0	10	0.936	0.313
**Conduct Problems**	2.21	1.562	0	8	0.851	0.649
**Hyperactivity**	3.17	2.056	0	10	0.482	−0.177
**Peer Problems**	1.51	1.648	0	10	1.499	2.677
**Prosocial Behavior**	7.13	1.941	0	10	−0.456	−0.047

Max.: Maximum; Min.: Minimum; SD: Standard Deviation.

**Table 2:** Centrality Metrics of the Overall Network.

Node	Betweenness	Closeness	Strength	Expected Influence
Reexperiencing	2	0.018	0.821	0.821
Avoidance	2	0.018	0.945	0.945
Increased Arousal	4	0.020	0.968	0.968
Emotional Problems	0	0.018	0.988	0.988
Conduct Problems	0	0.014	0.748	0.473
Hyperactivity	3	0.017	0.860	0.469
Peer Problems	0	0.015	0.561	0.352
Prosocial Behaviors	0	0.016	0.664	−0.211

## Data Availability

Due to ethical concerns, supporting data cannot be made openly available. Data and R script are available upon reasonable request.

## Supplementary Materials

The following supporting information can be downloaded at: https://www.mdpi.com/article/10.3390/ejihpe15110225/s1; Figure S1: accuracy of edge weights of the networks; Figure S2: differences between edges of the networks; Figure S3: centrality metrics stability indices; Figures S4 and S5: network graphs; Tables S1–S6: centrality indices of the networks.
