# Supplementary material for: Network Structure of Post-Traumatic Stress and Social/Emotional/Behavioral Difficulties in a Post-Earthquake Child and Adolescent Sample
Source: Eur J Investig Health Psychol Educ. 2025 Oct 31;15(11):225. doi: 10.3390/ejihpe15110225 (PMC12650814; doi:10.3390/ejihpe15110225)
Supplement: Supplementary file 1 [file ejihpe-15-00225-s001.zip › ejihpe-3800491-supplementary.pdf]

Figure S1

Accuracy of Edge Weights in the Overall Network.

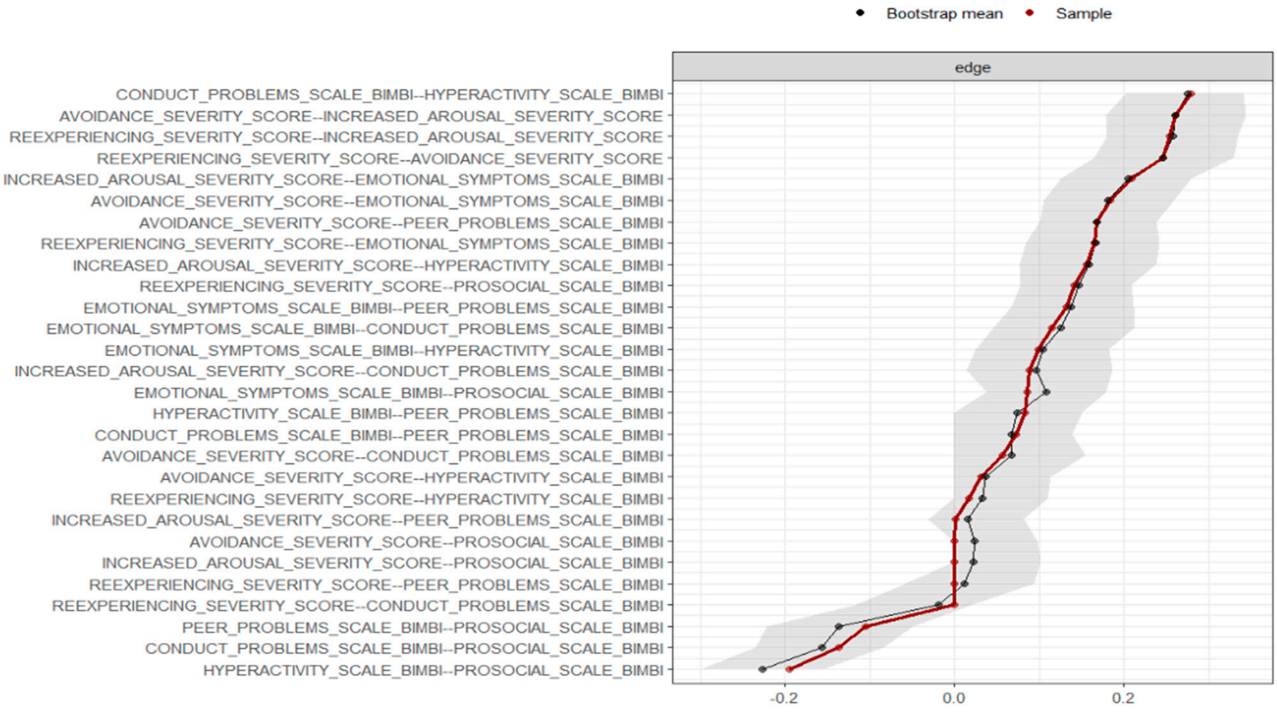

Note. Accuracy of edge weights in the network. The red line represents the sample edge weights, while the black dots indicate the bootstrap mean. The gray area represents the bootstrapped confidence intervals, indicating the variability of edge weight estimates. Narrower confidence intervals suggest more stable and reliable edges, whereas wider intervals indicate greater uncertainty in the edge estimation.

Figure S2

Bootstrapped Differences Between Edges in the Overall Network.

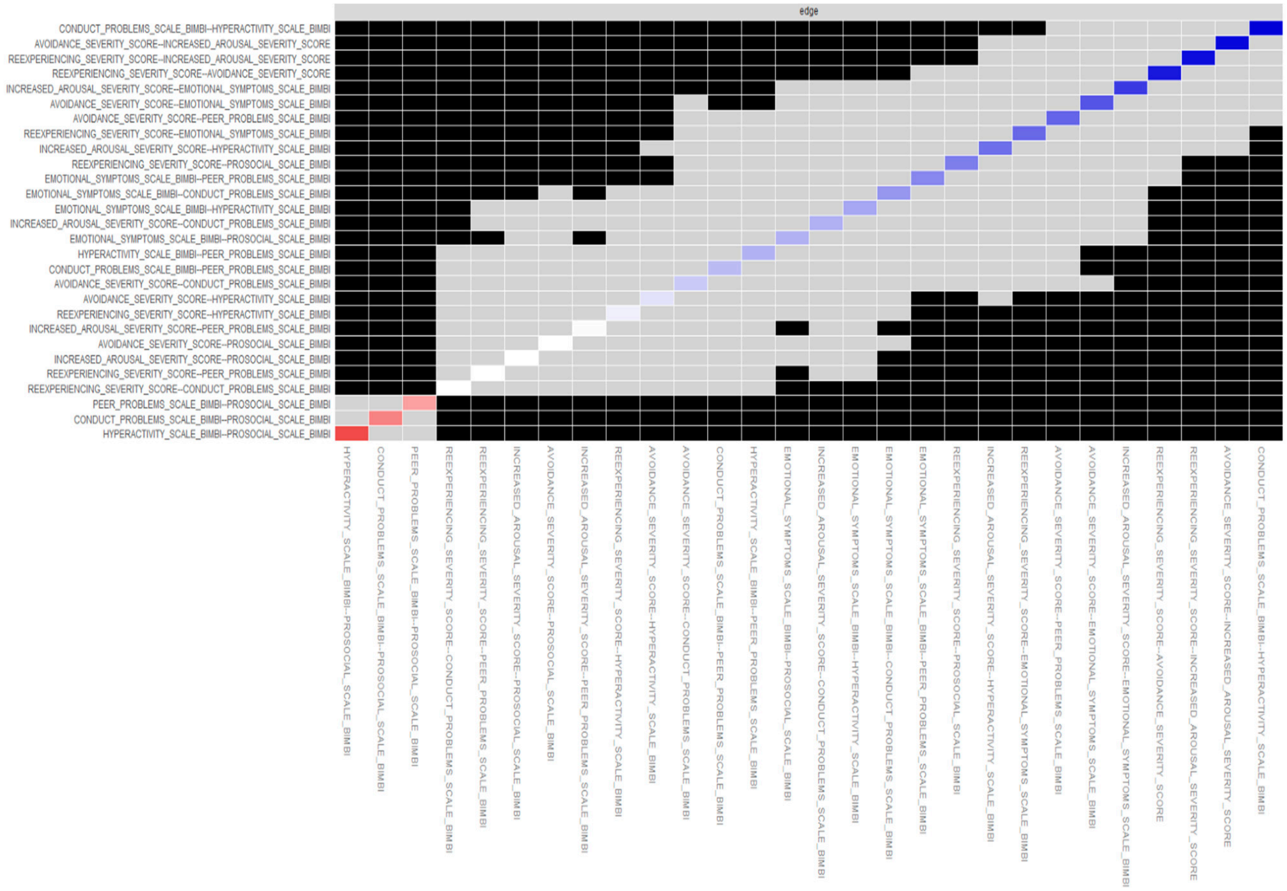

Note. Bootstrapped difference test for edge weights in the network. Gray boxes indicate edge weights that do not significantly differ from one another, while black boxes indicate edge weights that show statistically significant differences. Blue and red boxes on the diagonal correspond to edge weights with positive and negative correlations, respectively.

Figure S3

Coefficient Stability for Centrality Indices in the Overall Network.

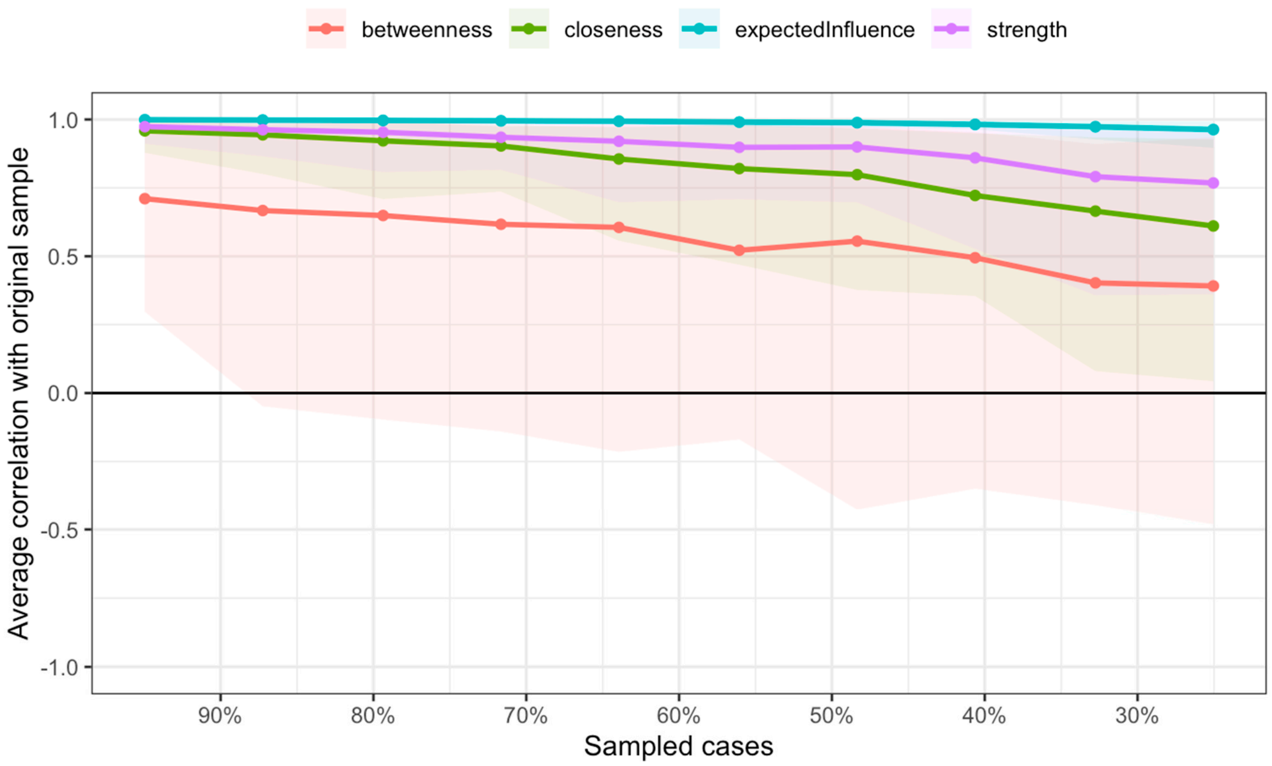

Note. Stability analysis of node centrality indices using case-wise resampling bootstrapping. The x-axis represents the proportion of sampled cases, while the y-axis shows the correlation with the original centrality values. Higher values indicate greater robustness of centrality measures. Values above 0.5 suggest acceptable stability, with Strength and Expected Influence showing the highest stability, while Betweenness appears more sensitive to sample variations.

Table S1

Centrality Indices of the Males Network.

Node	Betweenness	Closeness	Strength	Expected Influence
Re-experiencing	1	0.019	0.820	0.820
Avoidance	2	0.018	0.922	0.922
Increased Arousal	2	0.020	1.061	1.061
Emotional Symptoms	3	0.021	1.082	1.082
Conduct Problems	1	0.017	0.830	0.584
Hyperactivity	1	0.018	0.802	0.320
Peer Problems	1	0.018	0.759	0.336
Prosocial Behaviors	2	0.019	0.935	-0.215

Note. Centrality indices represent the relative (estimated) importance of each node in the network. The higher the value, the higher the relative (estimated) importance. Betweenness indicates the frequency with which the node is found in the shortest path between two other nodes; Closeness represents how close a node is to all other nodes in the network; Strength indicates how well a node is directly connected to other nodes; and Expected Influence, similarly to Strength, indicates how well a node is directly connected to other nodes, also taking the sign of edge weights into account.

Table S2

Centrality Indices of the Females Network.

Node	Betweenness	Closeness	Strength	Expected Influence
Re-experiencing	1	0.017	0.848	0.848
Avoidance	3	0.019	1.035	1.035
Increased Arousal	2	0.021	0.953	0.953
Emotional Symptoms	1	0.019	1.044	1.044
Conduct Problems	0	0.015	0.698	0.336
Hyperactivity	5	0.020	0.971	0.609
Peer Problems	0	0.014	0.418	0.371
Prosocial Behaviors	1	0.015	0.581	-0.190

Note. Centrality indices represent the relative (estimated) importance of each node in the network. The higher the value, the higher the relative (estimated) importance. Betweenness indicates the frequency with which the node is found in the shortest path between two other nodes; Closeness represents how close a node is to all other nodes in the network; Strength indicates how well a node is directly connected to other nodes; and Expected Influence, similarly to Strength, indicates how well a node is directly connected to other nodes, also taking the sign of edge weights into account.

Figure S4

Network of Symptoms in Primary School.

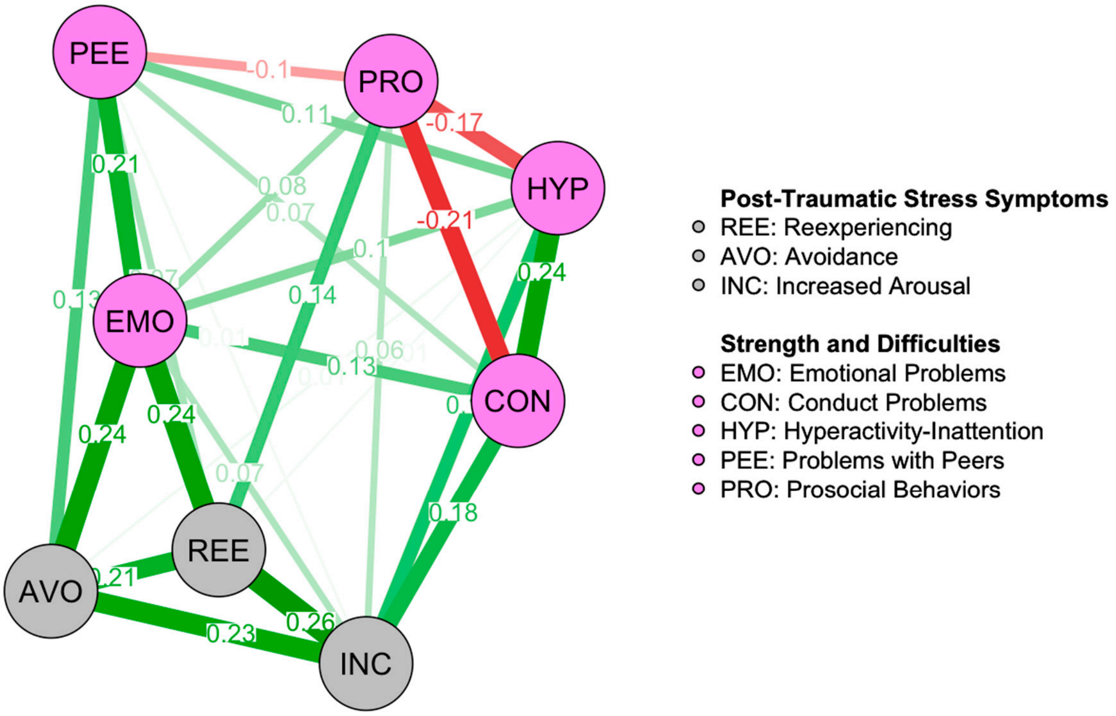

Note. Network model of PTSD symptoms and general psychopathology in primary school children exposed to the 2012 Emilia-Romagna earthquake. The model was regularized with the Extended Bayesian Information Criterion (EBIC) using Graphical Least Absolute Shrinkage and Selection Operator (EBICglasso). Green lines represent positive partial correlations between nodes (i.e., variables), while red lines represent negative partial correlations. Values reported on each line represent the partial correlation coefficient: higher values correspond to thicker lines. The sparsity of the network, indicating the proportion of zero-valued connections, is 0.107.

Table S3

Centrality Indices of the Primary School Network.

Node	Betweenness	Closeness	Strength	Expected Influence
Re-experiencing	3	0.021	0.935	0.935
Avoidance	1	0.018	0.818	0.818
Increased Arousal	4	0.020	0.959	0.959
Emotional Symptoms	2	0.020	1.071	1.071
Conduct Problems	1	0.019	0.821	0.403
Hyperactivity	0	0.017	0.787	0.437
Peer Problems	0	0.015	0.691	0.491
Prosocial Behaviors	0	0.016	0.769	-0.200

Note. Centrality indices represent the relative (estimated) importance of each node in the network. The higher the value, the higher the relative (estimated) importance. Betweenness indicates the frequency with which the node is found in the shortest path between two other nodes; Closeness represents how close a node is to all other nodes in the network; Strength indicates how well a node is directly connected to other nodes; and Expected Influence, similarly to Strength, indicates how well a node is directly connected to other nodes, also taking the sign of edge weights into account.

Figure S5

Network of Symptoms in Secondary School.

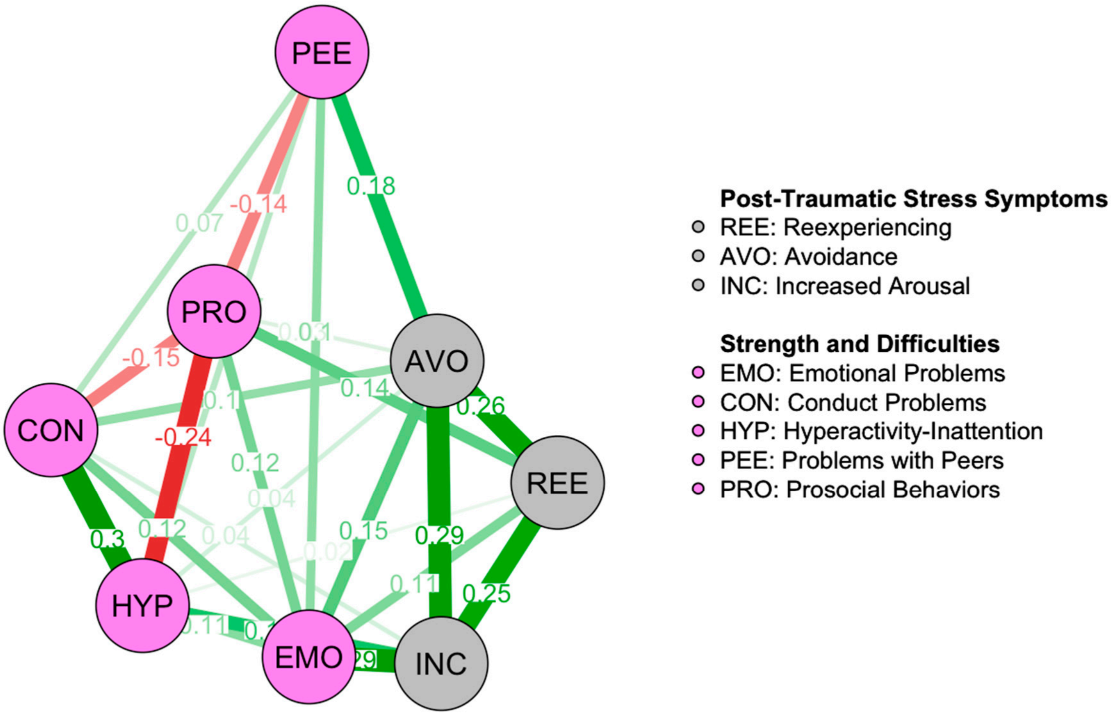

Note. Network model of PTSD symptoms and general psychopathology in secondary school students exposed to the 2012 Emilia-Romagna earthquake. The model was regularized with the Extended Bayesian Information Criterion (EBIC) using Graphical Least Absolute Shrinkage and Selection Operator (EBICglasso). Green lines represent positive partial correlations between nodes (i.e., variables), while red lines represent negative partial correlations. Values reported on each line represent the partial correlation coefficient: higher values correspond to thicker lines. The sparsity of the network, indicating the proportion of zero-valued connections, is 0.143.

Table S4

Centrality Indices of the Secondary School Network.

Node	Betweenness	Closeness	Strength	Expected Influence
Re-experiencing	1	0.018	0.790	0.790
Avoidance	2	0.020	1.054	1.054
Increased Arousal	4	0.022	1.033	1.033
Emotional Symptoms	0	0.019	0.999	0.999
Conduct Problems	0	0.015	0.773	0.480
Hyperactivity	3	0.019	0.937	0.461
Peer Problems	0	0.015	0.565	0.277
Prosocial Behaviors	2	0.018	0.819	-0.238

Note. Centrality indices represent the relative (estimated) importance of each node in the network. The higher the value, the higher the relative (estimated) importance. Betweenness indicates the frequency with which the node is found in the shortest path between two other nodes; Closeness represents how close a node is to all other nodes in the network; Strength indicates how well a node is directly connected to other nodes; and Expected Influence, similarly to Strength, indicates how well a node is directly connected to other nodes, also taking the sign of edge weights into account.

Table S5

Centrality Indices of the Network of Symptoms in Youth distant from the Epicenter.

Node	Betweenness	Closeness	Strength	Expected Influence
Re-experiencing	1	0.016	0.607	0.607
Avoidance	5	0.020	1.062	1.062
Increased Arousal	1	0.017	0.843	0.843
Emotional Symptoms	3	0.019	0.841	0.841
Conduct Problems	3	0.016	0.819	0.517
Hyperactivity	0	0.014	0.722	0.530
Peer Problems	1	0.015	0.516	0.256
Prosocial Behaviors	1	0.013	0.474	-0.280

Note. Centrality indices represent the relative (estimated) importance of each node in the network. The higher the value, the higher the relative (estimated) importance. Betweenness indicates the frequency with which the node is found in the shortest path between two other nodes; Closeness represents how close a node is to all other nodes in the network; Strength indicates how well a node is directly connected to other nodes; and Expected Influence, similarly to Strength, indicates how well a node is directly connected to other nodes, also taking the sign of edge weights into account.

Table S6

Centrality Indices of the Network of Symptoms in Youth closer to the Epicenter.

Node	Betweennes s	Closeness	Strength	Expected Influence
Re-experiencing	1	0.019	0.908	0.908
Avoidance	1	0.017	0.884	0.884
Increased Arousal	5	0.021	1.013	1.013
Emotional Symptoms	1	0.019	1.054	1.054
Conduct Problems	0	0.014	0.702	0.445
Hyperactivity	4	0.019	0.924	0.430
Peer Problems	0	0.015	0.572	0.382
Prosocial Behaviors	0	0.017	0.761	-0.179

Note. Centrality indices represent the relative (estimated) importance of each node in the network. The higher the value, the higher the relative (estimated) importance. Betweenness indicates the frequency with which the node is found in the shortest path between two other nodes; Closeness represents how close a node is to all other nodes in the network; Strength indicates how well a node is directly connected to other nodes; and Expected Influence, similarly to Strength, indicates how well a node is directly connected to other nodes, also taking the sign of edge weights into account.
